# Short Range Pipe Guided Wave Testing Using SH0 Plane Wave Imaging for Improved Quantification Accuracy

**DOI:** 10.3390/s22082973

**Published:** 2022-04-13

**Authors:** Filip Szlaszynski, Michael J. S. Lowe, Peter Huthwaite

**Affiliations:** NDE Group, Department of Mechanical Engineering, Imperial College London, London SW7 2AZ, UK; m.lowe@imperial.ac.uk (M.J.S.L.); p.huthwaite@imperial.ac.uk (P.H.)

**Keywords:** non-destructive testing, plane wave imaging, synthetic focusing imaging, guided wave testing, SH0 mode, defect sizing, part-circumferential part-depth cracks

## Abstract

Detection and criticality assessment of defects appearing in inaccessible locations in pipelines pose a great challenge for many industries. Inspection methods which allow for remote defect detection and accurate characterisation are needed. Guided wave testing (GWT) is capable of screening large lengths of pipes from a single device position, however it provides very limited individual feature characterisation. This paper adapts Plane Wave Imaging (PWI) to pipe GWT to improve defect characterization for inspection in nearby locations such as a few metres from the transducers. PWI performance is evaluated using finite element (FE) and experimental studies, and it is compared to other popular synthetic focusing imaging techniques. The study is concerned with part-circumferential part-depth planar cracks. It is shown that PWI achieves superior resolution compared to the common source method (CSM) and comparable resolution to the total focusing method (TFM). The techniques involving plane wave acquisition (PWI and CSM) are found to substantially outperform methods based on full matrix capture (FMC) in terms of signal-to-noise ratio (SNR). Therefore, it is concluded that PWI which achieves good resolution and high SNR is a more attractive choice for pipe GWT, compared to other considered techniques. Subsequently, a novel PWI transduction setup is proposed, and it is shown to suppresses the transmission of unwanted *S*0 mode, which further improves SNR of PWI.

## 1. Introduction

Cracks and corrosion in inaccessible locations in pipelines present a great challenge for the petrochemical and power industries. Inspection techniques which allow for remote defect detection and accurate characterisation are needed.

Guided Wave Testing (GWT) is one of the more recent non-destructive testing (NDT) methods for long range screening of tubular structures [1,2]. GWT is generally used to cover large lengths of pipes typically tens to hundreds of metres from a single device position, which makes this method a good candidate for the inspection of inaccessible locations. The information about a defect obtained from GWT inspection can give some indication of severity, since, in the simplest sense, a larger defect will cause a larger reflection amplitude. However, for defect circumferential extents smaller than 10–15%, it is difficult to distinguish between a defect which is narrow and deep from that which is wider and shallow [3]. This makes assessing whether the defect is critical or not a challenge [4]. The desired large volumetric coverage of GWT comes at the price of decreased sensitivity and very limited individual defect characterisation capabilities. Therefore, follow-up inspections on areas where defects are indicated are usually carried out locally using more accurate methods, e.g., ultrasonic testing [3]. These follow-up inspections of inaccessible locations are difficult, expensive and often require disruptive dismantling of structures.

### 1.1. Synthetic Focusing Imaging Methods

GWT with improved defect characterisation would be an attractive technique for stand-alone inspection of hard-to-reach structures. In bulk wave ultrasonic testing, defect detection and individual characterisation are often based on phased array synthetic focusing imaging [5,6,7,8]. In recent years, the two most popular high resolution imaging algorithms are Total Focusing Method (TFM) [9] and Plane Wave Imaging (PWI) [10,11,12].

In TFM, the image is reconstructed from Full Matrix Capture (FMC) acquired data [9]. By focusing both on transmission and reception for each pixel of the image, good reconstruction quality and high spatial resolution is achieved. The performance advantages of TFM come at the price of acquisition time and a large amount of data to process—the technique requires N transmission-reception cycles, where N is the number of elements in the array.

Lately, Plane Wave Imaging (PWI) has been the subject of increasing research interest [11,12,13,14,15]. The technique reconstructs the region of interest from signals backscattered after insonification of the inspected area with the plane waves sent at a chosen range of angles. The final compounded image is obtained by coherent addition of the images reconstructed by applying focus on reception for each plane wave angle. Le Jeune et al. (2016) [11] and Velichko and Croxford (2018) [16] have shown that TFM and PWI methods produce images of comparable resolution and quality, with fewer transmission-reception cycles required for PWI. Fewer transmissions means shorter acquisition time and less data to process. Hence, PWI is a very attractive method for fast and accurate high resolution imaging. Excitation of the probing plane wave using all the array elements per each emission provides much higher acoustic power compared to TFM, consequently, the inspection range in attenuating materials is better and the method is less sensitive to incoherent noise [11].

### 1.2. Synthetic Focusing Imaging in Pipe Guided Wave Testing

Synthetic focusing was first introduced to pipe GWT by Hayashi et al. (2005) [17]. Subsequently, the performance of the Common Source Method (CSM), the Synthetic Aperture Focusing Technique (SAFT) [18] and TFM was evaluated and compared for short range pipe guided wave imaging applications [4,19,20,21]. SAFT and ‘golden standard’ TFM were expected to outperform the CSM in terms of reconstructed image quality, however, the images of both of these methods were corrupted with coherent noise bands due to the *S*0 mode which is excited simultaneously with the desired torsional T(0,1) mode. Consequently, CSM was deemed the most suitable for pipe GWT applications due to its excitation of pure T(0,1). Its performance was evaluated in [4] and these results will serve for validation in this paper.

Despite the great potential for GWT, both SAFT and TFM suffer from coherent noise issues which significantly decrease their Signal to Noise Ratio (SNR). Furthermore, both techniques use a single insonifying element per acquisition, resulting in a probing wave of low acoustic power, hence reducing the effective inspection range in real-world applications, where high attenuation is often the problem. While CSM provides superior coherent SNR and higher acoustic power, its maximum achievable resolution is significantly lower than TFM due to focusing performed only on reception. Consequently, it provides inferior defect characterisation compared to TFM, which hinders limited access applications. Therefore, a technique capable of achieving high resolution while maintaining good SNR is needed.

This paper aims to address this problem, firstly through adaptation of the temporal bulk wave PWI algorithm for pipe GWT which has not been done before. Then, a filter removing undesired coherent noise from the FMC signals used for TFM reconstructions will be developed. This will be followed up by the evaluation of the performance of PWI against the chosen defect example and by its comparison to similar TFM and CSM deployments. A novel transduction set-up will also be proposed for deployment of guided wave PWI in pipes. It achieves suppression of the unwanted fundamental *S*0 mode hence improves coherent SNR. By addressing the known limitations of synthetic focusing techniques while maintaining comparable resolution, PWI has the potential to improve detection and characterisation of specific candidate defects (i.e., cracks) in pipes and other tubular structures. This is particularly desired in the GWT of inaccessible locations where the follow up inspection with any complimentary technique is not feasible, hence the assessment of defect severity has to predominantly rely on guided wave technology.

## 2. Adaptation of Time Domain Synthetic Focusing Imaging Algorithms to Pipe Guided Wave Testing

The synthetic focusing imaging techniques can be implemented in either time [9,11] or wavenumber–frequency (f-k) domain [22,23,24]. This paper uses the time domain heuristic delay-and-sum beamforming implementation of these algorithms, which is conceptually simpler and more suitable for restricted circumferential access. This implementation is an option with better stability, flexibility to arbitrary geometries and better performance against material inhomogeneities.

The adaptations of synthetic focusing imaging to pipe GWT are traditionally based on simplification of the analysis of the pipe guided waves to analogical analysis of plate guided waves in an unwrapped periodic plate [4,25,26,27]. This is achieved by approximating the set of pipe guided modes into a number of SH0 plate guided waves, neglecting the curvature of the pipe and implementing a boundary condition to match one side of the domain to the other, effectively unrolling the pipe.

This paper proposes a new, different approach to guided wave synthetic focusing imaging. The focus here is on short range GWT, which allows further simplification of the guided wave problem by looking at ‘local’ (plate) solutions, instead of ‘global’ (pipe) solutions. The proposed adaptation is also based on plate/pipe guided wave analogy. However, here the solutions do not have to be continuous around the pipe circumference, and hence there is no need for the extra boundary condition. The pipe is treated as a continuous infinite plate in which the angles for transmission and back propagation of SH0 mode are not restricted to discrete pipe-guided wave mode angles. This reduces the number of modes considered for imaging to a single fundamental shear horizontal mode, and hence simplifies the algorithm.

### 2.1. PWI Adaptation to Pipe GWT

**Figure 1:**
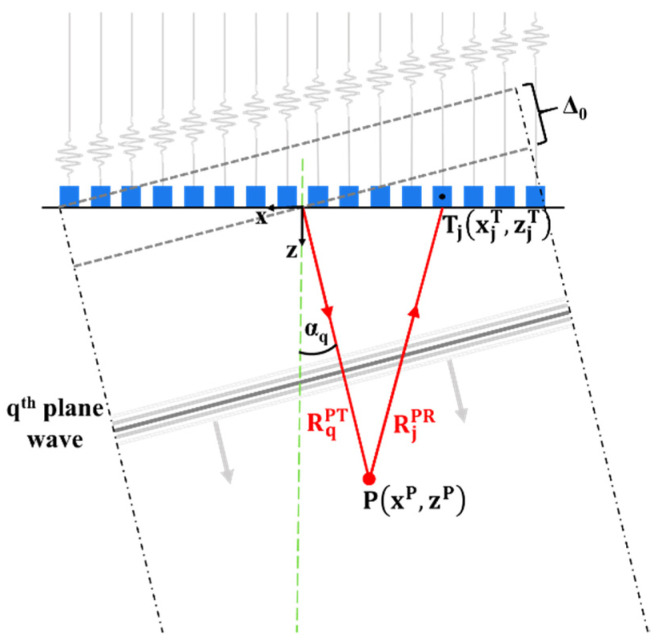
The conceptual outline of classic 2D PWI method. $R_{q}^{PT}$ is the qth incident plane wave travel path to the focus point P, $R_{j}^{PR}$ is the travel path on reception from P to jth array element T_j_, angle $\alpha_{q}$ is the plane wave angle and $\Delta_{0}$ is the correction factor offsetting the excitation to begin at t = 0.

Classic 2D PWI, outlined in Figure 1, reconstructs images from the Q × N matrix of backscattered signals m_qj_(t) recorded on all the elements of the N transducer array during Q transmission events. In a single transmission event q, all the array elements are excited to generate a plane wave propagating at an angle $\alpha_{q}$. The desired angle of propagation is achieved via application of appropriate delay laws. The response recorded after insonification of the region of interest with the qth plane wave is used for synthetic focusing on each pixel of the image on the reception, in the same manner as in the TFM algorithm. The reconstructions obtained for a range of Q angles are then coherently added to create the final PWI compound image. The intensity of the reconstructed pixel P($x^{P}$,$\;z^{P}$) can be mathematically expressed as [11]:

(1) $$I\left(P\right)=\left|\overset{Q}{\underset{q=1}{\sum }}\overset{N}{\underset{j=1}{\sum }}h_{qj}^{PWI}\left(\frac{R_{q}^{PT}+R_{j}^{PR}}{c}\right)\right|,$$

where $R_{q}^{PT}$ is the qth incident plane wave travel path from the transducer array to the arbitrary focus point P, $R_{j}^{PR}$ is the travel path on reception from the arbitrary focus point to the jth receiving element of the transducer array, $h_{qj}^{PWI}$ is the direct Hilbert transform of the m_qj_(t) signals and c is the wave velocity in the material.

**Figure 2:**
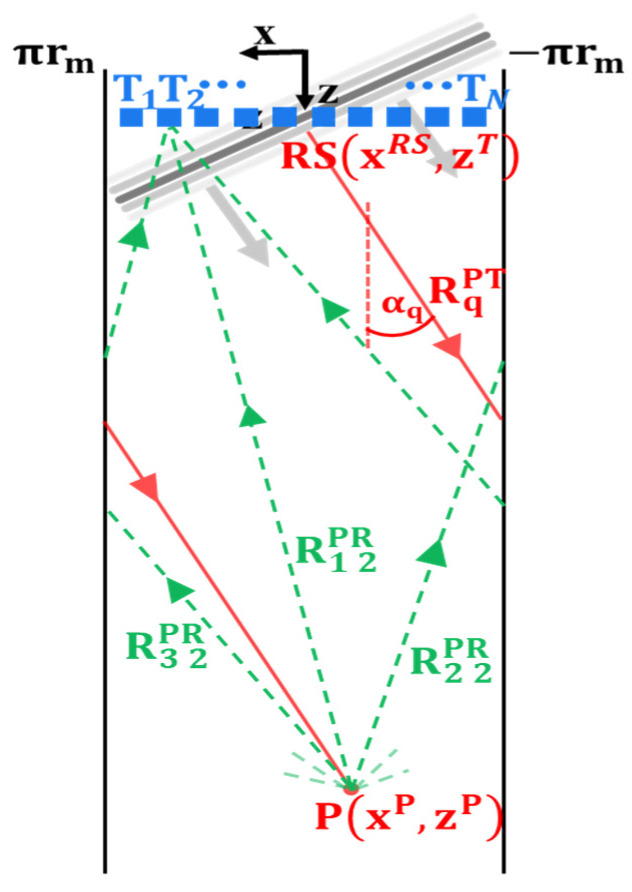
Schematic diagram of pipe PWI showing helical paths along which guided waves are travelling to and from a focusing point P for a qth plane wave send at an angle $\alpha_{q}$. RS is the start of the incident wave travel path to the arbitrary point P, $r_{m}$ is the mean radius of the pipe, $R_{mj}^{PR}$ is the travel path from P to the jth transducer T_j_ via m^th^ order helical path and $R_{q}^{PT}$ is the qth incident plane wave travel path to the focus point P.

The main difference between the 2D PWI and its pipe implementation is that the pipe wall is a closed geometry. The waves on transmission and reception travel around the pipe in helical paths as presented in Figure 2.

As a consequence of using signal contributions that have travelled through the pipe via different helical paths, the final reconstructed image in the pipe implementation of PWI is a coherent summation of reconstructions assuming each helical path. Therefore, an additional summation has to be added to the classic PWI Equation (1) to describe image intensity of pixel P in pipe guided wave PWI, as presented in Equation (2):

(2) $$I\left(P\right)=\left|\overset{M}{\underset{m=1}{\sum }}\overset{Q}{\underset{q=1}{\sum }}\overset{N}{\underset{j=1}{\sum }}h_{qj}^{PWI}\left(\frac{R_{q}^{PT}+R_{mj}^{PR}}{c}\right)\right|,\;$$

where M is the number of helical paths used for reconstruction and $R_{mj}^{PR}$ is the travel path from the focus point to the jth transducer via m^th^ order helical path (see Figure 2 for helical path numbering). $R_{mj}^{PR}$ is given by:

(3) $$R_{mj}^{PR}=\sqrt{{\left(-{\left(-1\right)}^{m}\times \left|x^{P}-x_{j}^{T}\right|+\frac{m}{2}\times circ_{m}\right)}^{2}+{\left(z^{P}-z_{j}^{T}\right)}^{2},}$$

where $\left(x_{j}^{T},\;z_{j}^{T}\right)$ are the coordinates of jth array element $T_{j}$, $circ_{m}=2\pi r_{m}$ is mean circumference of the pipe, $r_{m}$ is the mean radius of the pipe and m is the order of helical path.

**Figure 3:**
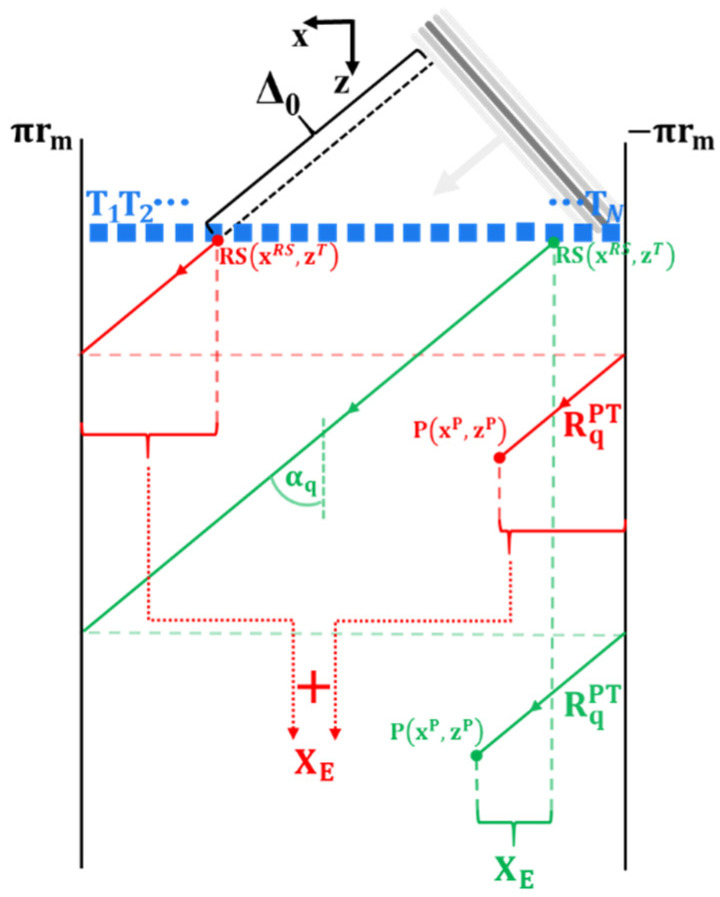
Outline of two cases of incident plane wave travel paths to focusing point P for a qth plane wave sent at an angle $\alpha_{q}$. $R_{q}^{PT}$ is the incident wave travel path to P, RS is the start of this path at the array, array element T_j_ is jth array element, $r_{m}$ is the mean radius of the pipe, *X_E_* is the length of the incident travel path projection in the circumferential direction minus the full pipe circumference repetitions accounting for the number of times the signal travelled around the pipe before reaching pixel P, and $\Delta_{0}$ is the correction factor offsetting the excitation to begin at t = 0.

Similarly to the travel paths on reception, the incident plane waves on transmission travel in the helical paths along the pipe. The helical path angle with the axis of the pipe depends on the plane wave angle $\alpha_{q}$. The incident wave travel path $R_{q}^{PT}$ can be expressed as:

(4) $$R_{q}^{PT}=\frac{z^{P}-z^{T}}{\cos\alpha_{q}}+\Delta_{0},$$

where *z^T^* is the axial position of the transducers $\left(z^{T}=z_{1}^{T}=z_{2}^{T}=\cdots =z_{N}^{T}\right)$ and $\Delta_{0}$ is the correction factor offsetting the plane wave excitation to begin at t = 0. Δ_0_ can be geometrically found as:

(5) Δ0={(xRS−xNT)sinαq    for         αq<0(xRS−x1T)sinαq    for         αq≥0,

where $x^{RS}$ is the x coordinate of the start of the incident wave travel path to the focusing point. For the ease of calculation, it is assumed that a continuous plane wave forms immediately at the transducer array. $x^{RS}$ can be expressed as:

(6) $$x^{RS}=\{\begin{array}{c} \begin{array}{ccc} \mathrm{sgn}\left(\alpha_{q}\right)\left(2\pi r_{m}-X_{E}\right)+x^{P}\;\;\;\; & when\;\;\; & X_{E}>\pi r_{m}+\mathrm{sgn}\left(\alpha_{q}\right)x^{P} \end{array}\\ \begin{array}{ccc} x^{P}-\mathrm{sgn}\left(\alpha_{q}\right)X_{E}\;\;\;\;\;\;\;\;\;\;\;\;\; & when\;\;\; & X_{E}\leq \pi r_{m}+\mathrm{sgn}\left(\alpha_{q}\right)x^{P} \end{array} \end{array},$$

where *X_E_* is the relative position in the x (circumferential) direction of the pixel P with respect to the starting position of the incident ray path (RS in Figure 2) through which the incident wave travels to this point P. Therefore, *X_E_* is effectively the length of the incident travel path projection in the circumferential direction minus the full pipe circumference repetitions accounting for the number of times the signal travelled around the pipe before reaching pixel P. There are two possible cases of *X_E_* outlined in Figure 3 with red and green colours. Mathematically, *X_E_* can be expressed as:

(7) $$X_{E}=\left|\left(z^{P}-z^{T}\right)\tan\alpha_{q}\right|-f_{c},$$

where $f_{c}=⎣\frac{\left|\tan\alpha_{q}\left(z^{P}-z^{T}\right)\right|}{2\pi r_{m}}⎦2\pi r_{m}$ subtracts full pipe circumference repetitions.

Unlike classic 2D PWI, where only a single direct ray path on reception from the focus point to the jth receiving element of the transducer array needs to be considered, here, in theory, there are infinite direct ray paths. All the backscattered signals from any point in the pipe will eventually reach the fully circumferential transducer array. This results in a virtually infinite aperture of the fully circumferential array. Additionally, each incident plane wave covers the entire interrogated area. This prevents the situation where the steering angle of a plane wave is out of range of the geometrical angles between the end array elements and an arbitrary reconstruction point which could lead to artefacts in the final image [13,16]. All this makes the pipe a powerful platform for performance comparison of synthetic focusing imaging techniques, as recording the full angular range of backscattered waves allows the highest possible resolution to be achieved [16]. In practice however, SH0 has limited scattering angles [21] and higher order helical path signals tend to contain more coherent and incoherent noise with respect to potential defect signals, which degrades the image quality. Therefore, the selection of maximum helical path order used for reconstruction is a fine balance between the increase in resolution and decrease in SNR. Therefore, finding the optimal imaging parameters for the best image quality and fair comparison of imaging methods is covered in Appendix A.

CSM is a special case of PWI with only a single 0° plane wave used for reconstruction hence Equation (2) is also used for CSM reconstruction. It is currently the most popular technique for pipe guided wave synthetic focusing imaging, because of its simplicity and great coherent and incoherent SNR [19]. Hence, CSM results will be treated as a baseline for comparison of PWI results and other methods in the present study.

### 2.2. *S*0-Cancelling Guided Wave PWI Transduction Setup

The torsional T(0,1) guided wave mode has been the most popular mode in pipe GWT commercial applications [1,4,28] thanks to its nondispersive nature and that it is not affected by liquid presence in the pipe. It is usually excited using shear contact transducers [29]. This type of transducer simultaneously excites two modes—the desired SH0 mode, which makes up the T(0,1) pipe guided wave mode, and an unwanted *S*0 guided wave mode in the direction parallel to the SH0 particle motion. Unwanted modes are a form of coherent noise. Their presence can result in artefacts and decreased SNR in the reconstructions.

Generation of the incident wave containing only a single desired wave mode is very challenging. This is overcome in the CSM method by using a single plane wave at 0°, but at the cost of decreased resolution to around 1–1.5 wavelengths, compared to the 0.5 wavelengths theoretically achievable by TFM and PWI. While research attention has been devoted to suppression of the resulting coherent noise in pipe guided wave TFM (e.g., [30]), PWI has not yet been implemented in pipe GWT. Here, the focus is on development of a novel PWI transduction setup which allows *S*0 suppression.

**Figure 4:**
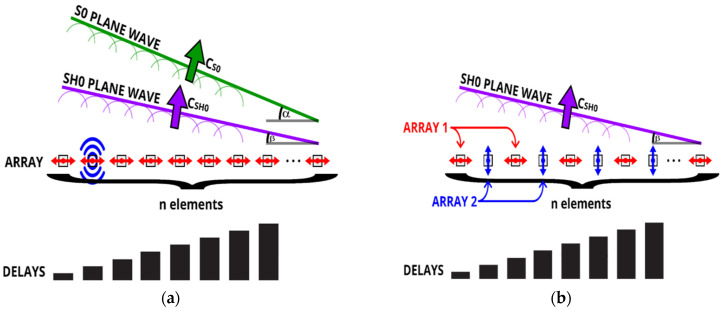
SH0 guided wave mode plane wave excitation using (**a**) classic transduction setup, and (**b**) *S*0 mode cancelling transduction setup.

Simple application of delay laws to excite the SH0 plane wave at an angle β using array elements oriented in the circumferential direction inevitably creates an additional unwanted *S*0 plane wave at angle α as illustrated in Figure 4a. The unwanted plane wave can result in artefacts in the reconstructed images because the *S*0 mode travels around 70% faster than SH0 mode. Adding a second array in which elements oriented in the axial direction are positioned between the elements of the primary transducer array, as outlined in Figure 4b, provides capability for modal control. The secondary array, which transmits signals 180° out of phase with respect to the primary array elements, is used to excite the *S*0 wave. When the appropriate amplitude weighting coefficient is established for the secondary array, it cancels out the unwanted *S*0 plane wave inevitably excited by the primary array. This weighting coefficient can be found by vector analysis and simplified to:

(8) $$w=Re\left(\tan\alpha_{S0}\right),$$

where the angle of the *S*0 plane wave is given as:

(9) $$\alpha_{S0}=\sin^{-1}\left(\frac{\Delta t\;c_{S0}}{d_{p}}\right),$$

∆t is the time between excitation of the consecutive array elements given by delay laws, $d_{p}$ is pitch distance and $c_{S0}$ is the *S*0 wave speed. The SH0 mode excited in the circumferential direction by the secondary array does not constructively interact to create the shock wave within the array angular transmission range nor can it be received on elements of the primary array oriented circumferentially. The *S*0 suppression algorithm is only needed for an SH0 angular range between –36.6° and 36.6° because beyond this angle range the real part of $\alpha_{S0}$ is 90° and hence the weighting coefficient is zero.

### 2.3. TFM Adaptation to Pipe GWT

TFM can also be adapted to pipe GWT using the simplified pipe-plate analogy. In FMC acquisition array elements can usually be treated as omnidirectional point sources for simplicity. This means that the incident wave propagates in a wide range of angles forming a circular pattern around the source. Signals travel via different helical paths inclined at a range of angles between arbitrary point P and ith transmitting and jth receiving array elements. The intensity of the reconstructed pixel P($x^{P}$,$\;z^{P}$) can be calculated using the following equation:

(10) $$I\left(P\right)=\left|\overset{M}{\underset{m_{T}=1}{\sum }}\overset{M}{\underset{m_{R}=1}{\sum }}\overset{N}{\underset{i=1}{\sum }}\overset{N}{\underset{j=1}{\sum }}h_{ij}^{FMC}\left(\frac{R_{m_{T}i}^{PT}+R_{m_{R}j}^{PR}}{c}\right)\right|,$$

where $R_{m_{T}i}^{PT}$, $R_{m_{R}j}^{PR}$ are transmission/reception travel paths from i^th^ transducer to reconstructed pixel and from pixel to jth transducer, respectively, and *M_T_* and *m_R_* are helical path orders on transmission/reception, respectively. The incident and scattered wave travel paths can be calculated using Equation (3) and their geometrical representation is analogical to helical paths on reception in PWI marked with a green colour in Figure 2.

As before, in theory, there are infinitely many helical paths to consider. However, practically, transducers are not omnidirectional and their true angular transmission/reception range is typically around –π/4 to π/4. This limits the number of helical paths usable for reconstruction. As for other imaging methods, there is also a trade-off between the resolution and SNR. Therefore, the optimal number of helical paths providing the best image quality is investigated in Appendix A to provide fair comparison of the imaging methods.

**Figure 5:**
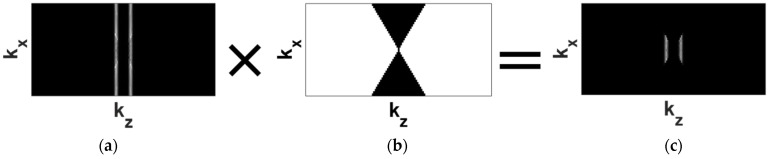
Spatial filtering of recorded time traces, which removes waves travelling at higher angles; (**a**) original signal after 2D FFT, (**b**) spatial frequency mask removing waves at angles larger than $\alpha_{cf}$ and (**c**) Fourier domain signal after filtering.

Unlike PWI, in TFM the angular range of transmitted waves cannot be conveniently controlled. To achieve comparable resolution and SNR to PWI, and to remove the circumferential guided wave artefacts mentioned in [19,30], the waves travelling at angles higher than the desired angular range need to be filtered out in the 2D spatial frequency domain (k-space) from the recorded FMC time traces. This can be achieved using the frequency mask removing spatial components of waves at angles larger than the desired cut-off angle $\alpha_{cf}$ as outlined in Figure 5. A gradual transition region between the full pass and total removal of the spatial frequency components is introduced to avoid sharp changes leading to strong ‘ringing’ artefacts [31].

### 2.4. Guided Wave Full Matrix Capture Plane Wave Imaging (FMCPWI)

Classic PWI is a method of data acquisition and a reconstruction algorithm. It is possible to synthesize signal datasets for PWI reconstruction algorithm from the data acquired using FMC acquisition by application of appropriate delays to each response matrix constituting the FMC dataset. This can be achieved via the following transformation:

(11) $$h_{qj}^{FMCPWI}=\{\begin{array}{c} \overset{N}{\underset{i}{\sum }}h_{ij}^{FMC}\left(t-\frac{\left(i-1\right)d_{p}}{c}\sin\left|\alpha_{q}\right|\right)\;\;\;\;\;\;\;\;when\;\;\;\;\;\;\;\;\alpha_{q}\geq 0\;\\ \overset{N}{\underset{i}{\sum }}h_{ij}^{FMC}\left(t-\frac{\left(N-i\right)d_{p}}{c}\sin\left|\alpha_{q}\right|\right)\;\;\;\;\;\;\;when\;\;\;\;\;\;\;\;\;\alpha_{q}<0 \end{array}.$$

In this paper, the PWI algorithm applied to a plane wave response dataset synthesised from the FMC data is referred to as FMCPWI to distinguish from the classic PWI implementation. It provides a convenient platform for comparison of TFM and PWI reconstruction algorithms as well as FMC and PWI data acquisition approaches. The reconstruction algorithm in FMCPWI remains unchanged from pipe guided wave PWI and is given in Equation (2).

## 3. Methodology—Simulation Model and Experimental Setup

The key performance indicators for imaging methods include spatial resolution and SNR. This paper focuses on lateral resolution which is the minimum distance at which two defects can be resolved (i.e., distinguished) from the reconstructed image. Ability to correctly size the defect is tightly related to resolution. When the crack is small with relation to the wavelength it acts as point scatterer and its indication is essentially a point spread function, which is often used to assess the resolution [9,13]. By measuring the length of the known defect from the reconstructed image and comparing results with true dimensions resolution can be estimated. Here, defect lengths were estimated using a full width at half maximum (FWHM) method, i.e., taking the size as being the distance between the two points where the image intensity has dropped to 50% of the peak [4]. The defect lateral size at which its indication in the reconstructed image starts to resemble a point scatter, i.e., indication size stops decreasing with further decrease of the lateral extent of a defect, reveals the maximum achievable resolution.

**Figure 6:**
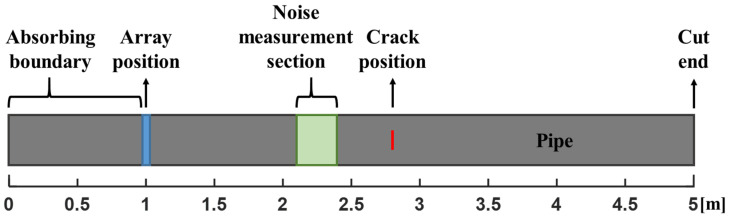
FE pipe model layout.

In the presented study SNR is defined as the ratio of the peak defect indication (I_PDI_) to the RMS background noise (I_NRMS_) in the section of the pipe preceding a defect between 1.1 m and 1.4 m (see Figure 6) with respect to axial position of an array (see Equation (12)). This specific area was chosen because it does not include any artefacts caused by reflections of other modes from a defect and does not include dead zone artefacts near the array in the experiments.

(12) $$SNR=20\log_{10}\frac{I_{PDI}}{I_{NRMS}}$$

### 3.1. Simulation Study

The numerical study was conducted using the Pogo Finite Element (FE) solver developed at Imperial College London [32]. The 3D FE model represented an 8 inch nominal diameter schedule 60 ASTM A106-B steel pipe later used in the laboratory experiment. The FE model (see Figure 6) had a structured mesh with minimum 30 elements per SH0 wavelength (λ_SH0_ ≈ 63.5 mm) in any direction. 12 elements were modelled through the pipe wall thickness. The selected element type was a general purpose linear brick element, with reduced integration (C3D8R). The pipe was modelled to be 5 m long with the phased array position offset from the pipe end by 1 m. The area between the array position and the closest cut end of the pipe was defined using absorbing elements, so it acted as an absorbing boundary [33]. Therefore, there were no waves propagating in the negative axial direction meaning that there were no spurious reflections introducing unwanted features into the image.

The 40 element fully circumferential ring phased array of equally spaced shear contact transducers was modelled by selecting nodes on the outer surface of the pipe which lie within the area approximately equal to the real contact area of the transducers in experiments. The ultrasonic waves were excited by application of force to these nodes in the tangential direction. The pitch distance was 17.3 mm, which is well below λ_SH0_/2 to ensure it is fully sampled so as to avoid grating artefacts.

Zero volume outer surface breaking planar cracks extending in the circumferential direction were modelled by disconnecting adjacent elements in the pipe wall at 1.8 m away from the phased array position [4,34]. Crack lengths from 2% (~0.2 λ_SH0_) to 40% (~4 λ_SH0_) of the pipe circumference were simulated to capture the relationship between defect size and accuracy of defect length estimation from the reconstructed images. The study was repeated for 25%, 50%, 75% and 100% crack through wall thickness depths.

The excitation signal was a 5 cycle Hann windowed tone burst with centre frequency of 50 kHz, the same tone burst was later used in experiments. The FE simulations include only signals and coherent noise; they do not include incoherent noise.

### 3.2. Experimental Setup

The 4 m long pipe used in the experiments (see Section 3.1. for pipe details) was chosen based on similarity to the one used in [4], which allows easy comparison and cross validation of some of the results. The SH0 velocity in the sample material was measured to be 3236.2 m/s.

**Figure 7:**
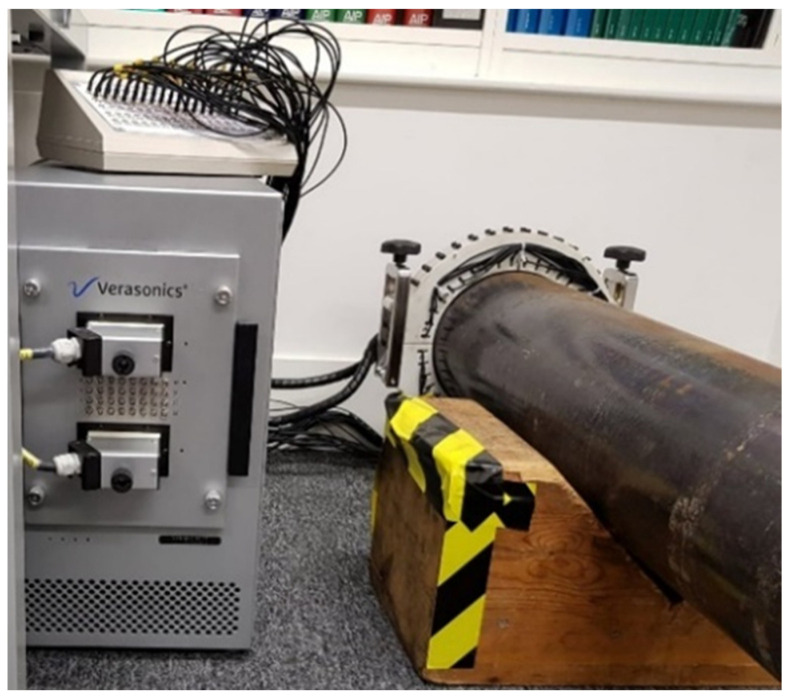
Experimental setup.

The design of the SH0 guided wave phased array ring used for the experiment was created with the help of Guided Ultrasonics Ltd. (Brentford, UK) [1]. The ring array, presented in Figure 7, consisted of two rows of circumferentially oriented shear contact transducers, each containing 40 elements. In the experiment, only a single row was used for transmission and reception, as there was no need for directional control. Two parts of the ring were screwed together on the top and bottom of the pipe to clamp it firmly onto the sample as presented in the experimental setup in Figure 7. The ring was placed at the cut end of the pipe so that the active row of transducers is placed right at the edge to suppress any waves travelling in the negative z-direction. An even contact between the transducers and pipe surface was provided by a spring-loaded mechanism at each element.

A Verasonics Vantage^TM^ 32LE phased array controller in a low frequency configuration was used for sending and recording the signal via the transducer ring. The sampling frequency on reception was 2.5 MHz. The recorded signal was filtered using the bandpass filter with a lower limit of 35 kHz and upper limit of 65 kHz.

**Figure 8:**
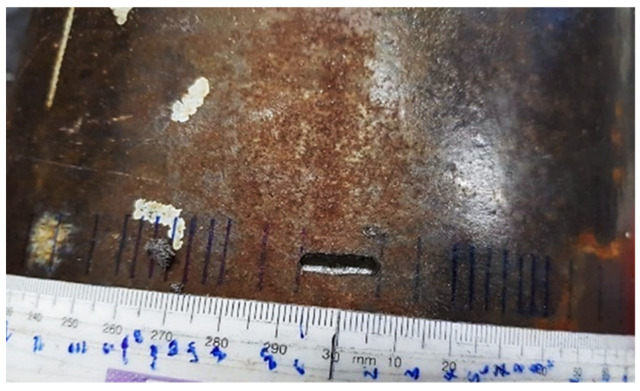
A 0.2 λ_SH0_ long circumferential notch used in the experiments.

Circumferential cracks were simulated via introduction of a 3 mm wide and 5.4 mm deep notch (50% of the wall thickness) at 1.8 m away from the array. The 3 mm axial extent of the notch was very small in comparison to the wavelength, hence the notch approximates a narrow crack well [34]. To validate the FE study, the length of the notch was progressively grown in the circumferential direction in small increments from 2% to 40% of the pipe circumference (see Figure 8) while the width and depth of the notch was kept constant. This way the FE findings on the relationship between crack length and sizing accuracy (resolution) could be validated experimentally by recreating the results for a 50% through thickness deep crack.

## 4. Results and Discussion

This section presents PWI results, compares them to CSM, TFM and FMCPWI and evaluates the performance of the novel PWI transduction setup with *S*0 cancelling. For the fair comparison of all four image reconstruction methods, they were implemented here using the optimal imaging parameters found in Appendix A. The comparison is based on FE and experimental results. The study evaluates the example case with a range of part-circumferential part-depth cracks/notches.

### 4.1. Lateral Defect Sizing Comparison

**Figure 9:**
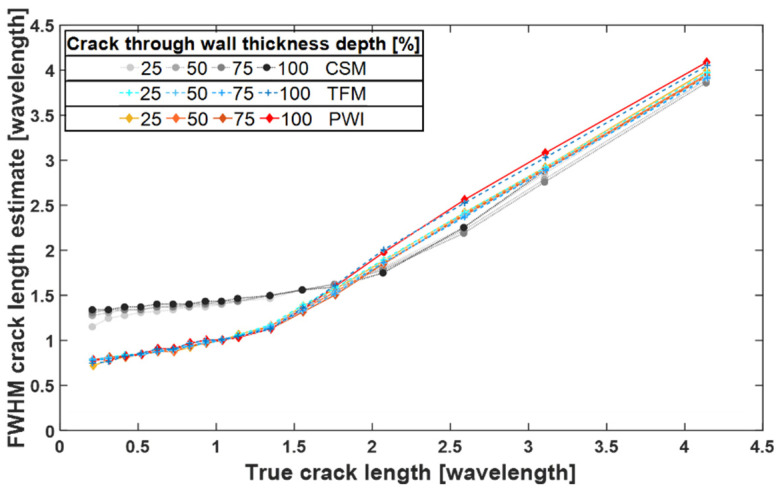
Comparison of FWHM sizing capabilities of CSM, TFM and PWI using FE simulations.

Figure 9 shows the comparison of the FWHM crack length estimate curves obtained from CSM, TFM and PWI.

From Figure 9, the maximum achievable resolution by SH0 CSM is around 1.4 λ_SH0_ which is consistent with results presented in [4], where the angular spectrum method implementation of CSM was used. These comparable results mean that the time domain implementation of synthetic focusing imaging algorithm yields the same resolution as the angular spectrum method. They also serve as cross validation for the FE study presented in this paper.

PWI and TFM crack length estimate curves look almost identical, and they plateau at the same value. This means that their maximum achievable resolution is comparable, and it is around 0.85 λ_SH0_, which is close to the theoretical 0.5 wavelength diffraction limit. This is a significant improvement in comparison to the maximum achievable resolution by CSM, therefore, both TFM and PWI are better suited for characterisation of small defects.

**Figure 10:**
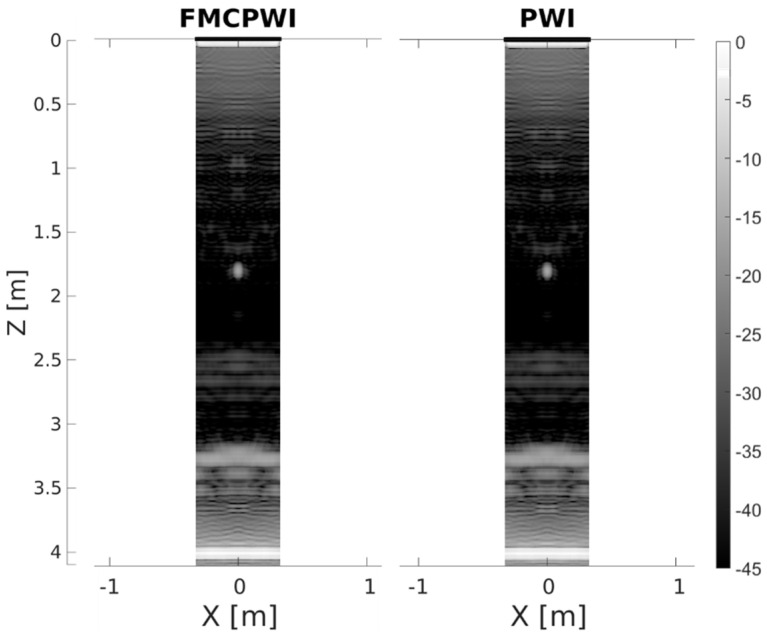
Comparison of FE FMCPWI (**left**) and FE PWI (**right**) reconstructions presented in the form of unwrapped pipe. X and z are circumferential and axial directions, respectively.

As presented in Figure 10, PWI and FMCPWI yield identical results in FE simulations. This means that the method of acquisition, FMC or transmission of actual plane waves, has no impact on resolution nor coherent SNR, as indeed should be expected. Its influence on incoherent SNR will be investigated in the next section.

**Figure 11:**
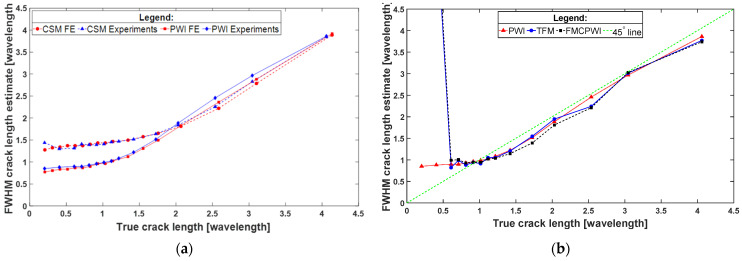
Comparison of FWHM sizing capabilities of (**a**) PWI and CSM using experimental and FE results for 50% through thickness deep notch/crack, (**b**) PWI, TFM and FCPWI using experimental results for 50% through thickness deep notch.

Figure 11a presents comparison of FE and experimental FWHM crack/notch length estimate curves obtained from CSM and PWI reconstructions. The curves obtained from the experiments closely resemble the ones from the FE study, which supports validity of the FE results. The experimental results confirm that PWI clearly outperforms CSM in terms of achievable resolution.

Figure 11b compares the FWHM notch length estimate curves for PWI, TFM and FMCPWI obtained from the experiments. As expected, all three methods perform alike for almost all notch lengths, except for short notches. In the case of notches shorter than 0.6 λ_SH0_, TFM and FMCPWI curves reveal great notch size overestimation, while PWI behaviour resembles the behaviour observed in the FE study. The severe notch length overestimation is a sign that the sizing algorithm does not distinguish notch indication from the noise related artefacts. This happens when the noise level is less than −6 dB below the defect indication amplitude. This makes sizing small defects unreliable. The error in defect sizing is given by deviation of the FWHM size estimate points from the 45° line of ideal sizing. For each method, within its resolution limits, the error is less than 10–15%, and typically much less than that, as can be seen in Figure 11b. It rapidly increases when the defect size is below the resolution limit.

### 4.2. SNR Comparison

According to the FE results presented earlier in Figure 9 and Figure 10, TFM, PWI and FMCPWI should produce comparable results with resolution on the same level and coherent SNR sufficient to reliably size small defects. Therefore, poor SNR in experiments is attributed to incoherent noise which is only present in experiments. In FE, PWI and FMCPWI results are indistinguishable, while in experiments PWI clearly suffers less from incoherent noise issues compared to FMCPWI. Since the only difference between these two techniques is the acquisition method, it can be concluded that the increased noise level is associated with the acquisition method. FMCPWI uses the same FMC data as the TFM algorithm and only these two methods experience SNR issues. Furthermore, the FMC data is filtered in the Fourier domain prior to TFM and FMCPWI reconstructions, hence if the noise was coherent, significant SNR improvement should be observed, however this is not the case.

**Figure 12:**
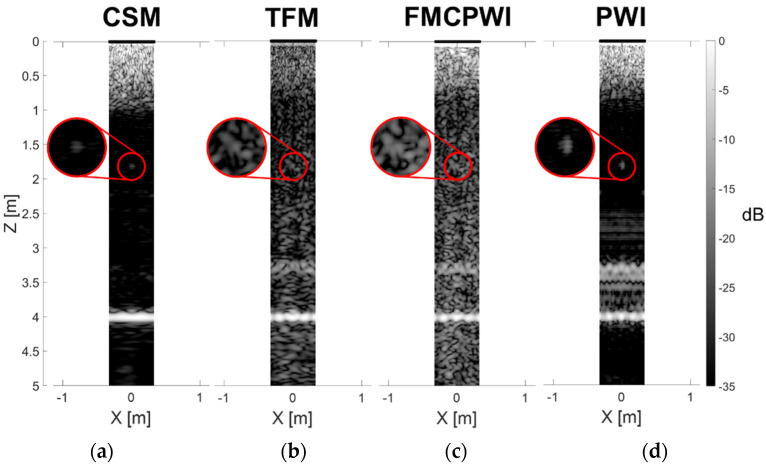
Comparison of unwrapped pipe reconstructions of 0.2 λ_SH0_ long, 50% through wall thickness deep notch at 1.8 m axial distance from the array using (**a**) CSM, (**b**) filtered TFM, (**c**) filtered FMCPWI and (**d**) PWI, from experimental data.

**Figure 13:**
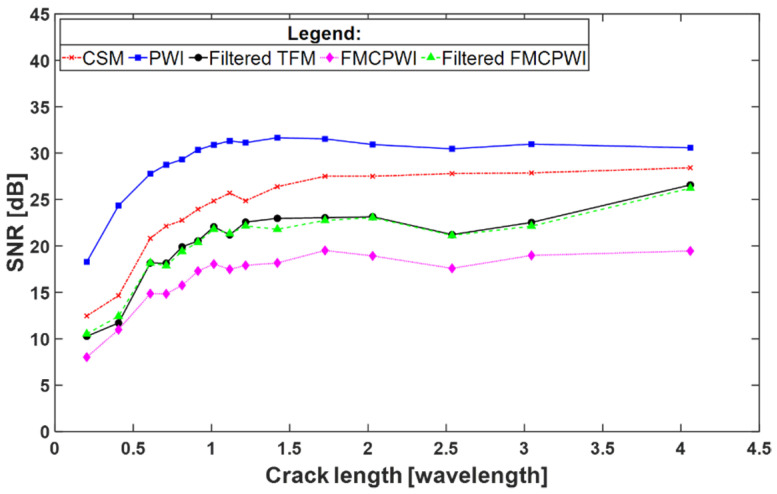
SNR vs. true crack length for 50% through wall thickness notch measured from CSM, PWI, filtered TFM, unfiltered FMCPWI and filtered FMCPWI reconstructions obtained from experimental data.

Figure 12 shows the reconstructions of a pipe with a 0.2 λ_SH0_ long, 50% through wall thickness deep notch at 1.8 m axial distance from the array obtained using all four studied synthetic focusing imaging methods. The empirical inspection of these images further supports the above hypothesis—one can notice in the TFM and FMCPWI reconstructions artefacts typical for incoherent noise. Moreover, for this short notch, TFM and FMCPWI have insufficient SNR to distinguish defect indication from the noise artefacts which hinders defect detection sensitivity of these methods. Figure 13 shows the comparison of SNR for all four considered techniques. It can be noted that, in general, FMC based methods have lower SNR in comparison to CSM and PWI. Counterintuitively, PWI achieves higher SNR compared to CSM. The latter technique uses only a single plane wave for reconstruction whereas PWI uses multiple acquisitions. Using multiple acquisitions for each reconstruction is in some respect equivalent to averaging and results in improved SNR of PWI in comparison to CSM. On the other hand, TFM uses multiple acquisitions, but this is not enough to compensate for very low transmission energy. Energy transmitted per each acquisition in FMC approach is only a fraction of energy transmitted during each PWI acquisition. As a consequence, TFM’s SNR is lower than the SNR of PWI or even CSM. FMCPWI and TFM using Fourier filtered FMC data achieve identical SNR. FMCPWI using unfiltered FMC data achieves on average around 5 dB lower SNR, compared to the filtered FMC implementations. However, even with Fourier filtering SNR of FMCPWI and TFM reconstructions are on average around 10 dB lower compared to reconstructions obtained from classic unfiltered PWI. This difference is associated with a difference in incoherent SNR between FMC and PWI methods of acquisition.

From the above results, it can be concluded that the performance of the techniques based on FMC is primarily limited by SNR. In experiments, PWI noticeably outperforms these techniques in terms of sensitivity to small defects. Despite resolution of FMCPWI and TFM being comparable to PWI, these techniques should not be deployed for detection and sizing of small defects without any methods mitigating incoherent noise such as averaging or coded excitation. While these mitigating methods provide improvement in SNR, they have some drawbacks which should be considered while choosing a technique for short range GWT inspection. The advantage of PWI is that it does not require application of these mitigating techniques, providing good SNR thanks to the high energy transmission per each acquisition. Research presented in [13,16] also suggests that PWI can achieve comparable resolution to TFM with fewer acquisitions. Hence, PWI is particularly attractive for arrays with large number of elements. Additionally, each PWI inspection automatically acquires data that can be used for classic T(0,1) guided wave inspection and to produce CSM reconstruction, which is advantageous.

### 4.3. PWI *S*0 Cancelling Transduction Setup

**Figure 14:**
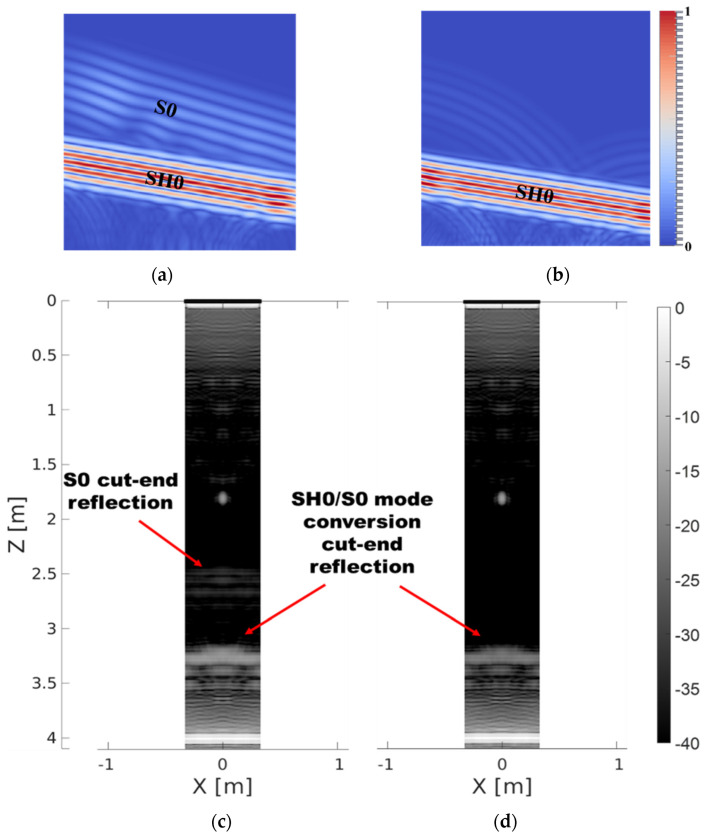
FE comparison of classic (**a**,**c**) and *S*0-cancelling (**b**,**d**) transduction setups. Simulation of transmitted 10° plane waves are illustrated at the top, and PWI reconstructions of pipe with 0.2 λ_SH0_, 75% through wall thickness deep crack at 1.8 m axial distance from the array are presented at the bottom. All four images are in the form of unwrapped pipe.

The new transduction setup cancelling the unwanted *S*0 mode which was proposed in Section 2.2 was implemented in FE simulation. Figure 14a,b shows the comparison of a transmitted 10° SH0 plane wave with and without *S*0 cancelling. It is apparent that the *S*0 cancelling setup suppresses the unwanted *S*0 plane wave that would be otherwise excited when applying the delay law to create a plane wave at a desired angle. There are only negligible residue *S*0 waves left from the first and last elements of the array.

The convenient way to quantify the reduction of *S*0 related artefacts in final reconstruction is to measure a drop of intensity of *S*0 reflection from the cut-end of the pipe, which results in a ‘fake’ cut-end echo in the image. Additionally, some of the energy of SH0 and *S*0 plane waves mode converts during reflection from the cut-end of the pipe. The SH0 mode converts to the *S*0 mode and *S*0 converts to SH0. The artefacts caused by both mode-converted waves appear in the same location in the reconstructed image due to reciprocity. Therefore, the suppression of *S*0 transmission should result in a noticeable reduction in the SH0-*S*0/*S*0-SH0 artefact as well. Figure 14c,d compares PWI reconstructions obtained using classic transduction and *S*0 cancelling transduction. It reveals that there is at least −20 dB reduction (from −30 dB to −50 dB) of the *S*0 ‘fake’ cut-end echo artefact to levels undistinguishable from the background noise level, which indicates complete *S*0 suppression. Furthermore, there is around −5 dB reduction (from around −16 dB to −21 dB) of SH0 to *S*0 and *S*0 to SH0 ‘fake’ cut-end artefact due to reduction of the *S*0 to SH0 contribution, because the *S*0 incident wave is completely suppressed. The biggest contribution to this artefact is from the SH0 to *S*0 mode conversion, which is unavoidable. Apart from reduction of unwanted artefacts in the reconstructed images, the *S*0 suppression method does not have a negative impact on resolution as the number of transmitting/receiving SH0 elements oriented in the circumferential direction is the same. Therefore, if there is a need to eliminate all the artefacts caused by illumination with *S*0 plane wave, the presented method could be implemented without negative impact on quality of the reconstructions.

## 5. Conclusions

This paper adapted time domain implementation of Plane Wave Imaging (PWI) to pipe guided wave testing and compared it to other time domain implementations of popular synthetic focusing imaging techniques such as Common Source Method (CSM) and Total Focusing Method (TFM). The key performance indicators were lateral resolution and signal-to-noise ratio (SNR). The comparison of imaging techniques was based on FE and experimental studies.

The experimental results supported validity of the FE findings. The key conclusions were that PWI and TFM achieve comparable resolution which is superior to CSM resolution. The FMC based methods achieve a substantially lower SNR compared to methods involving plane wave acquisition (PWI and CSM). Consequently, TFM and PWI implementation using synthesized FMC data were insensitive to smaller notches in experiments, indicating that these techniques should not be deployed without incoherent noise mitigation methods, while PWI and CSM achieved sufficient SNR to detect the smallest introduced notches, PWI achieves better resolution with significantly higher overall SNR making it a more attractive choice for pipe GWT. Additionally, a novel PWI transduction setup was proposed and evaluated in an FE study. It suppresses the transmission of unwanted *S*0 mode in PWI and hence reduces some coherent noise artefacts further improving the overall SNR.

## Figures and Tables

**Figure A1:**
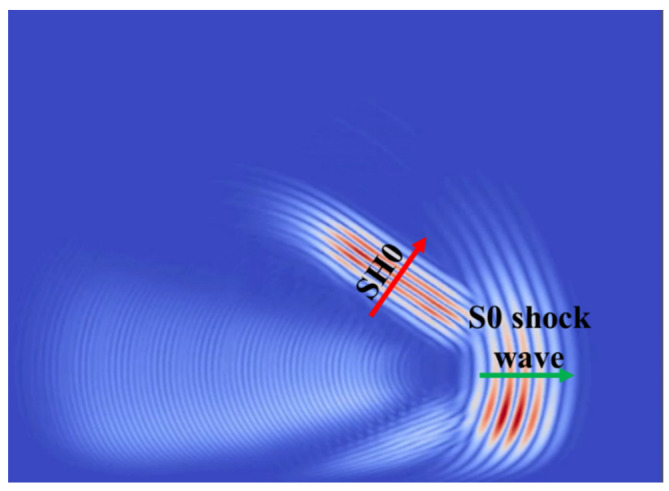
Unwrapped pipe schematic presenting *S*0 shock wave appearing at 36.6° SH0 plane wave transmission angle.

**Figure A2:**
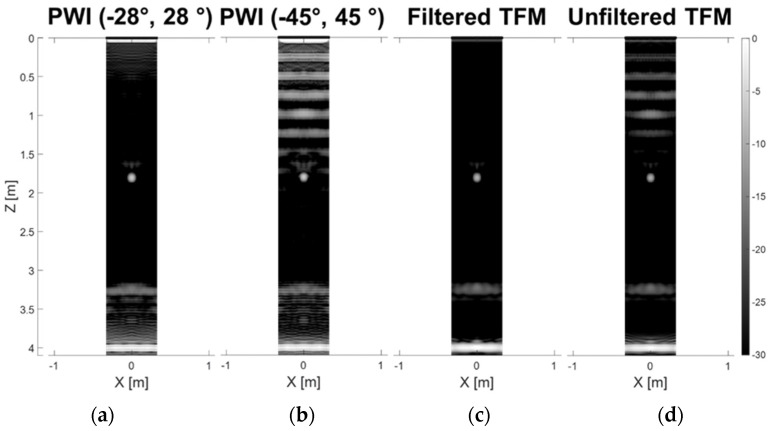
Comparison of reconstructions of FE pipe model with 1.3 λ_SH0_, 75% through thickness deep crack at 1.8 m axial distance from the array. The unwrapped pipe images were reconstructed using PWI with the transmission angular range of (**a**) −28° to 28°, (**b**) −45° to 45°, and TFM using (**c**) FMC with waves travelling at angles larger than 28° removed, and (**d**) unfiltered FMC data. The image intensity is in decibel scale with respect to the cut-end reflection.

**Figure A3:**
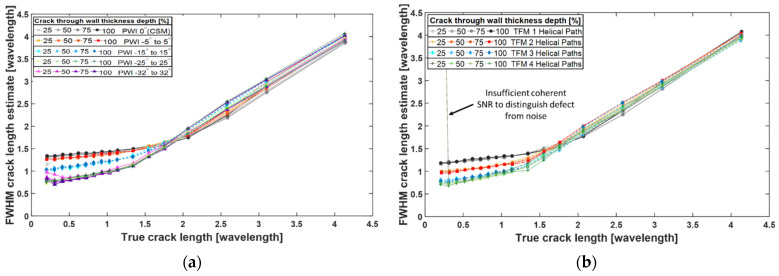
Plot of crack length estimates against true crack lengths measured from (**a**) PWI reconstructions using different plane wave transmission angular ranges, and (**b**) TFM reconstructions for different range of helical paths used for synthetic focusing on transmission. Based on FE simulations.

**Figure A4:**
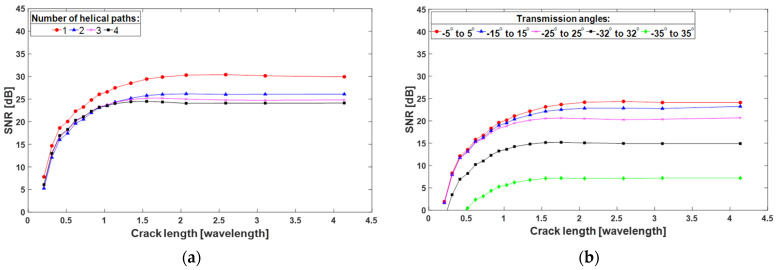
Coherent SNR vs. true crack length measured from (**a**) TFM and (**b**) PWI reconstructions with different angular ranges used on transmission. Based on FE simulations of 50% through wall thickness crack.

**Figure A5:**
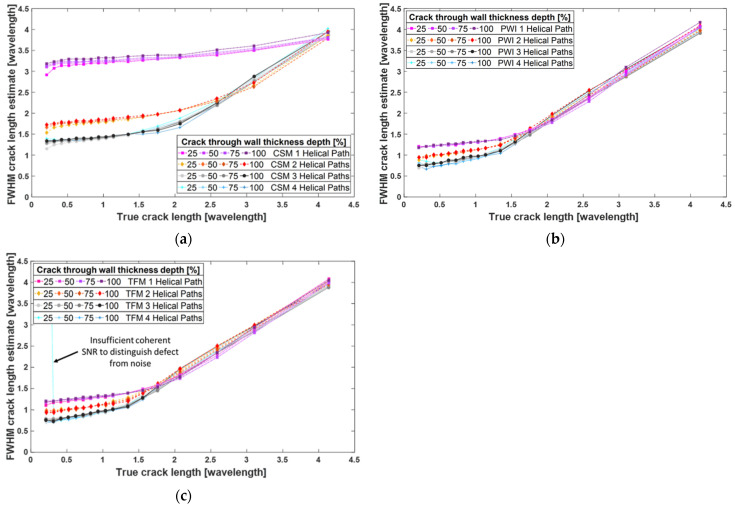
Crack length estimates obtained from (**a**) CSM, (**b**) PWI and (**c**) TFM reconstructed images from FE data, plotted against true crack lengths for different range of helical paths used for focusing on reception.

**Figure A6:**
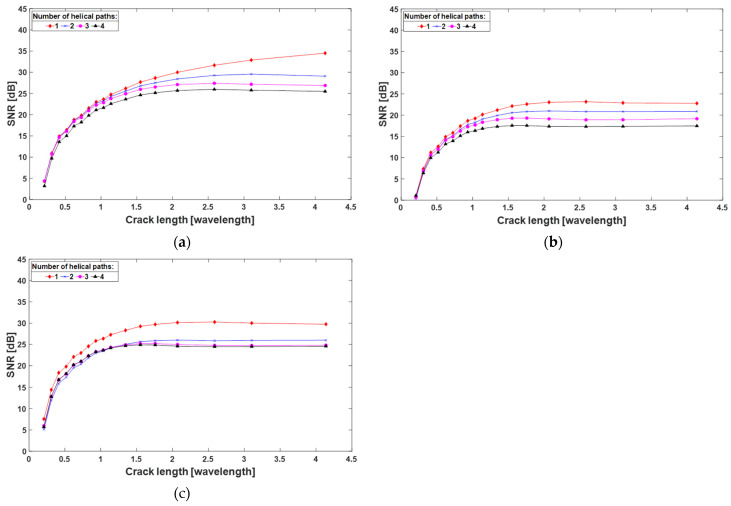
Coherent SNR vs. true crack length from (**a**) CSM, (**b**) PWI and (**c**) TFM reconstructions. Based on FE simulations of 50% through wall thickness crack.

## Data Availability

The data presented in this study are available on request from the corresponding author. The data are not publicly available due to the large size of datasets.

## Appendix A. Selection of Optimal Imaging Parameters

Here, we use FE simulations to find the optimal imaging parameters for each of the synthetic focusing imaging methods, so that they can be compared fairly. The imaging methods were optimized for resolution and coherent SNR. The FMCPWI was omitted here because the influence of imaging parameters on resolution and SNR is identical as in PWI. In PWI, the angular step between the transmitted plane waves was set to be 1°, which according to [13] is sufficient to achieve maximum resolution.

### Appendix A.1. Transmission

First, it was identified that PWI reconstructions suffer from the same coherent *S*0 noise bands as TFM does (e.g., [19]). In PWI, these appear due to excitation of a shock wave, portrayed in Figure A1. It occurs when the interval between the excitation of subsequent array elements, needed to achieve desired angle αq, closely matches the time, which takes for the *S*0 guided wave mode (excited as a by-product) to travel the pitch distance between the array elements. Consequently, excitation of every subsequent element adds energy to the circumferentially propagating wave front gradually increasing its amplitude.

The critical SH0 angle at which the shock wave occurs can be found from the following equation:

(A1) $$\alpha_{crit.}=\sin^{-1}\left(\frac{c_{SH0}}{c_{S0}}\right),$$

In the case study presented in the paper, this critical angle was 36.6°. Avoiding PWI transmission angles around this critical angle eliminates the unwanted *S*0 artefacts as presented in Figure A2a,b. Given the analogy between FMC and PWI acquisition methods [16], to avoid the coherent noise bands in TFM reconstructions, angles around the same critical angle should be filtered out, for example, using the method outlined in Section 2.3 Figure A2c,d show the comparison of TFM reconstruction using unfiltered data to a reconstruction with waves travelling at angles larger than 28° filtered out from the FMC data. Further investigation showed that filtering does not affect defect indication and has no negative impact on achieved resolution, therefore TFM using filtered FMC data was used for all reconstructions throughout this paper.

Next, the influence of the range of angles used for focusing on transmission on resolution and SNR was investigated. In PWI, the angular range of physically excited waves was varied, whereas in TFM the same was achieved via adjusting the number of helical paths used for synthetic focusing on transmission. In this section, the number of helical paths used for synthetic focusing on reception was kept constant at 3 helical paths for all the considered imaging methods. Figure A3 compares sizing capabilities of (a) PWI with transmission angles from 0° (CSM) to −32° to 32°, and (b) TFM with 1 to 4 helical paths used for synthetic focusing on transmission. Only angles up to −32° to 32° were included in PWI to avoid coherent noise issues mentioned in the previous paragraph.

Figure A3a,b show that there is improvement in resolution with the increase in the transmission angular range used for reconstructions in both techniques. In TFM, the noticeable improvement in resolution is between 1 and 3 helical paths. There is marginal improvement using 4 helical paths, however, the coherent SNR is insufficient to distinguish defects from noise for very small cracks. This limits detectability of small defects, hence 3 helical paths is preferred. In PWI, the angular range between −28° to 28° was found to be the range beyond which there is no further improvement in resolution in the case study presented in this paper.

Subsequently, the influence of angular range on SNR was investigated. The SNR curves for TFM and PWI are plotted in Figure A4a,b, respectively.

It can be seen in Figure A4a,b, that in general, there is a decrease in SNR with an increase in angular range used for focusing. In PWI, there is a considerable drop in SNR when increasing transmission angular range beyond −25° and 25°. This is because the *S*0 noise band artefacts are increasingly appearing when approaching the critical angle of 36.6°. The observed negative SNR for shorter cracks means that the RMS of coherent noise is higher than the defect indication amplitude, which is not desired. Therefore, to avoid coherent noise issues transmission angles should be kept well below the critical angle. In TFM, on the other hand, the considerable SNR drop is not observed when increasing angular range on transmission and coherent SNR appears to be around 5 dB higher compared to PWI. This is caused by the filtering used on FMC data, which removes the coherent noise beyond the desired angular range. Comparable results would be achieved if there was no filtering used in TFM (see Figure A2).

### Appendix A.2. Reception

To select the optimal angular range on reception, its influence on image resolution and SNR was investigated. For each FE model, images of the interrogated area were reconstructed using the range of helical path orders from 1 to 4. Estimated crack lengths were plotted against their true values for each imaging method. In this section, the angular range of transmitted plane waves in PWI was kept constant at −28° to 28° and in TFM 3 helical paths were used for focusing on transmission. The FWHM crack length estimate plots for CSM, PWI and TFM are presented in Figure A5.

In Figure A5, for all three methods, with the increase in the number of helical paths from 1 to 3, the FWHM curves plateau at significantly lower crack lengths for each additional helical path of higher order used for reconstruction of the image. Therefore, a wider angular range on reception results in better resolution. There is no change when increasing the number of helical paths from 3 to 4, or further. The relationship between the angular range used for reconstructions and sizing accuracy obeys the law of diminishing returns, i.e., when the angular range is small, increase in that range results in significant improvement in resolution, but as the angular range gets wider, its further broadening yields less impact on the achieved resolution. Eventually the angular range reaches the point at which its further increase does not improve resolution, which is the maximum achievable resolution.

Figure A6 presents the SNR values for reconstructed images using a different range of helical path orders for 50% crack through thickness depth.

In Figure A6, the same pattern can be observed for all three imaging methods—SNR noticeably degrades with increase in number of helical paths used for reconstruction. As expected, CSM has higher coherent SNR in comparison to PWI and TFM, thanks to 0° excitation effectively suppressing the unwanted modes.

### Appendix A.3. Selection of Parameters

From the above results, it can be concluded that achieving optimal image reconstruction resolution and coherent SNR depends (amongst other factors) on appropriate selection of angular ranges on transmission and reception. The angular range used for reconstruction has to be sufficient to allow the best resolution, however extending it beyond this range decreases coherent SNR and hence degrades the quality of the image.

In the case study presented in this paper, the optimal number of helical paths on reception to achieve the best defect reconstruction at 1.8 m away from the array was found to be 3 for all the considered imaging methods. For PWI and FMCPWI, the optimal transmission range was found to be between −28° to 28° and for TFM the optimal number of helical paths used for focusing on transmission was 3.

It is worth noting that to achieve the same optimal resolution throughout the whole length of the pipe in the time domain implementation of synthetic focusing imaging, the number of helical paths used for reconstruction varies with axial position. In general, in classic bulk wave imaging, resolution decreases with axial distance from the array as the effective transmission/reception angular range decreases away from the array. In the case of pipe GWT, when using a set number of helical paths throughout the length of the pipe the same phenomenon occurs. This can be mitigated by progressively increasing the number of helical paths used for reconstruction of pixels with increase of axial distance to maintain quasi constant angular range on reception. In other words, pixels further away would have to be reconstructed using more helical paths than pixels closer to the array. In practice, for short range pipe applications, a fixed number of helical paths is typically sufficient. In PWI, transmission angles remain constant throughout the length of the pipe.
